# Preso regulates NMDA receptor-mediated excitotoxicity via modulating nitric oxide and calcium responses after traumatic brain injury

**DOI:** 10.1038/s41419-019-1731-x

**Published:** 2019-06-24

**Authors:** Peng Luo, Xin Li, Xiuquan Wu, Shuhui Dai, Yuefan Yang, Haoxiang Xu, Da Jing, Wei Rao, Hongyu Xu, Xiangyu Gao, Zhou Fei, Hongbing Lu

**Affiliations:** 1 Department of Neurosurgery, Xijing Hospital, Fourth Military Medical University, Xi’an, China; 2 Department of Biomedical Engineering, Fourth Military Medical University, Xi’an, China; 3 Department of Anesthesiology, Xijing Hospital, Fourth Military Medical University, Xi’an, China

**Keywords:** Brain injuries, Brain injuries, Trauma, Trauma

## Abstract

Traumatic brain injury (TBI) has become a major health concern worldwide, and the poor outcome of TBI increases the need for therapeutic improvement. Secondary injuries following TBI, including excitotoxicity, lead to synaptic dysfunction and provide potential targets for intervention. Postsynaptic scaffold proteins, which are involved in the regulation of excitotoxicity after neuronal injury, play a crucial role in modulating synaptic function. Therefore, exploring the role of postsynaptic scaffold proteins in TBI might uncover new treatments. In this study, we demonstrated that downregulated expression of the postsynaptic scaffold protein Preso protects against neuronal injury after TBI in vitro and in vivo, and these effects are related to the inhibition of N-methyl-D-aspartate receptor (NMDAR) function. Further study showed that Preso facilitates signaling from NMDAR to nitric oxide (NO) and calcium (Ca^2+^) responses. First, the complex constituting NMDAR, postsynaptic density-95 (PSD-95), and neuronal nitric oxide synthase (nNOS) was shown to be involved in the Preso regulation of the NO response. Uncoupling the linkage between Preso and PSD-95 attenuated the stability of this complex and suppressed the regulatory effect of Preso on the NO response. In addition, phosphorylation of NMDAR by cyclin-dependent kinase 5 (CDK5) was shown to be responsible for the Preso-mediated Ca^2+^ response, which was dependent on the interaction between Preso and CDK5. These results suggested that the association of Preso with NMDAR signaling can serve as a target for neuroprotection against TBI.

## Introduction

Traumatic brain injury (TBI) is caused by external forces on the head, which cause closed or penetrating head injury and brain dysfunction. Due to its high mortality and morbidity, TBI has become a worldwide public health problem and substantially burdens society^1^. Excitotoxicity is one of the most important mechanisms underlying the pathophysiological process of TBI. Activation of glutamate receptors by overproduction of glutamate at synapses contributes to the formation of excitotoxicity. Therefore, inactivation of glutamate receptors, including the N-methyl-D-aspartate receptor (NMDAR) and the metabotropic glutamate receptor (mGluR), by pharmacological antagonists has exhibited neuroprotective effects in animal experiments. However, these potential drugs did not show satisfactory curative effects on TBI patients in a series of clinical trials^2^.

Postsynaptic scaffold proteins are a special group of molecules belonging to postsynaptic density (PSD) proteins, which directly connect glutamate receptors with their postsynaptic signaling pathways^3^. Homer proteins are key postsynaptic scaffold proteins that regulate mGluR signaling via interactions with mGluR and its downstream mediators in the pathogenesis of TBI. The postsynaptic density-95 (PSD-95) protein is another important postsynaptic scaffold protein that binds NR2B, a subunit of NMDAR, together with neuronal nitric oxide synthase (nNOS). Recently, pharmacological inhibitors that uncouple PSD-95 from NMDAR signaling have been shown to reduce trauma-induced brain damage and neurological deficits^4^. These results indicated that postsynaptic scaffold proteins are potentially valuable for investigating the mechanism underlying the pathogenesis of TBI and potential targets for TBI interference.

PSD-95-interacting regulator of spine morphogenesis (Preso) is a novel scaffold protein that contains PSD-95⁄Dlg⁄ZO1 (PDZ); 4.1, ezrin, radixin, and moesin (FERM); and WW domains^5^. Preso reportedly interacts with mGluR and Homer via its FERM domain and Homer ligand site to coordinate the function of mGluR by enhancing mGluR-Homer binding^6^. Furthermore, Preso binds with PSD-95, providing a link for NMDAR. Through these interactions with glutamate receptors, Preso regulates dendritic outgrowth and excitatory synaptic transmission and is further involved in the regulation of excitotoxicity^7^. Because of the crucial role of postsynaptic scaffold proteins in TBI, Preso provides a potential target for interfering with TBI. However, the role of Preso in TBI and how glutamate receptor signaling pathways are integrated for the Preso-dependent modulation of neuronal injury after TBI have not been fully elucidated. In this study, we showed that Preso, a novel postsynaptic scaffold protein, modulated NMDAR-related excitotoxicity after TBI. First, downregulation of Preso expression inhibited NMDAR-induced neurotoxicity and improved neuronal survival and recovery in models of TBI. Second, the effects of Preso on TBI were associated with its regulation of NO and Ca^2+^ responses induced by NMDAR. Third, Preso modulated NMDAR function after TBI by influencing the stability of the NR2B/PSD-95/nNOS complex and NR2B phosphorylation.

## Results

### Dominant positive effects of Preso on traumatic neuronal injury

**Fig. 1:**
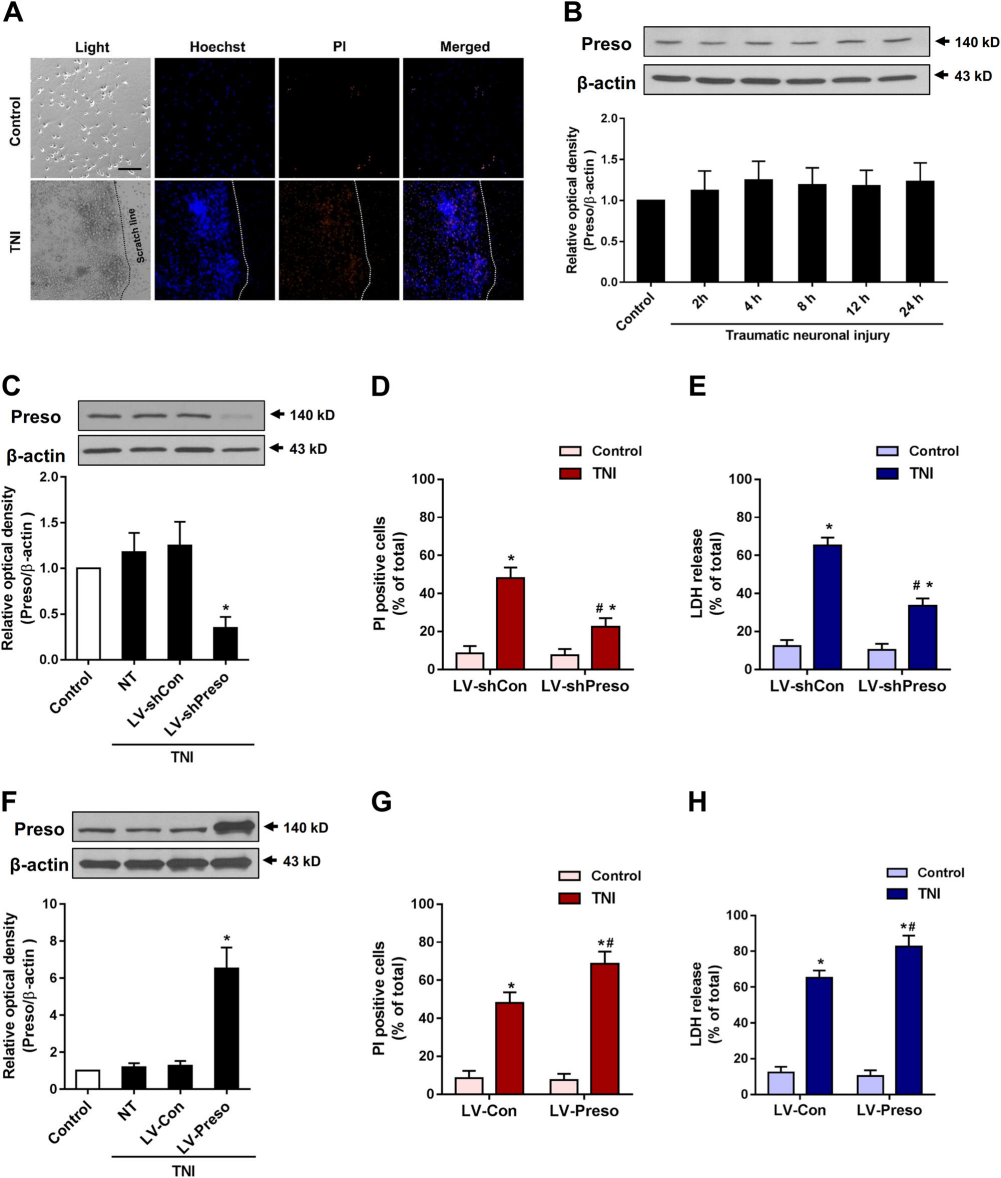
Preso contributes to neurotoxicity after traumatic neuronal injury. Traumatic neuronal injury (TNI) was induced in mouse neuronal cultures for 24 h. The representative images of neuronal culture at 12 h after TNI were obtained from light microscopy and PI/Hoechst staining (**a**). Scale bar = 100 μm. The protein expression of Preso was analyzed by western blot (**b**). The data are presented as the mean ± SEM from five experiments. After transfection of LV-shCon and LV-shPreso and TNI, the protein expression of Preso was analyzed by western blot (**c**), the cell death rate was assessed by PI/Hoechst staining (**d**), and the cytotoxicity was determined by an LDH assay (**e**). The data are presented as the mean ± SEM from five experiments. *p < 0.05 *vs*. control and ^#^*p* < 0.05 *vs*. LV-shCon. After transfection of LV-Con and LV-Preso and TNI, the protein expression of Preso was analyzed by western blot (**f**), the cell death rate was assessed by PI/Hoechst staining (**g**), and the cytotoxicity was determined by an LDH assay (**h**). The data are presented as the mean ± SEM from five experiments. *p < 0.05 *vs*. control and ^#^*p* < 0.05 *vs*. LV-Con

To investigate the effects of TBI on Preso, cortical neurons were used to establish a traumatic neuronal injury (TNI) model, an in vitro model of TBI (Fig. 1a). Preso expression was assessed by immunoblot at different time points. Preso expression remained stable after TNI (Fig. 1b). Then, lentiviral transduction was used to determine the role of Preso in neuronal survival and death after TNI (Fig. S1). Immunoblot analysis showed that a lentiviral-expressed shRNA targeting Preso (LV-shPreso) significantly decreased the expression of Preso (Fig. 1c), and a Preso-targeted lentivirus (LV-Preso) significantly increased the expression of Preso (Fig. 1f). Propidium iodide (PI) staining showed that LV-shPreso transfection reduced cell death at 12 h after TNI, while LV-Preso transfection exerted the opposite effect on neuronal cell death (Fig. 1d, g). In addition, a lactate dehydrogenase (LDH) release assay was used to analyze the cytotoxicity of neurons at 12 h after TNI. As shown in Fig. 1e, downregulation of Preso expression obviously decreased the release of LDH from neurons after TNI, while increased LDH release from neurons was observed after upregulation of Preso expression (Fig. 1h).

### Preso modulated NMDAR-related excitotoxicity after TNI

**Fig. 2:**
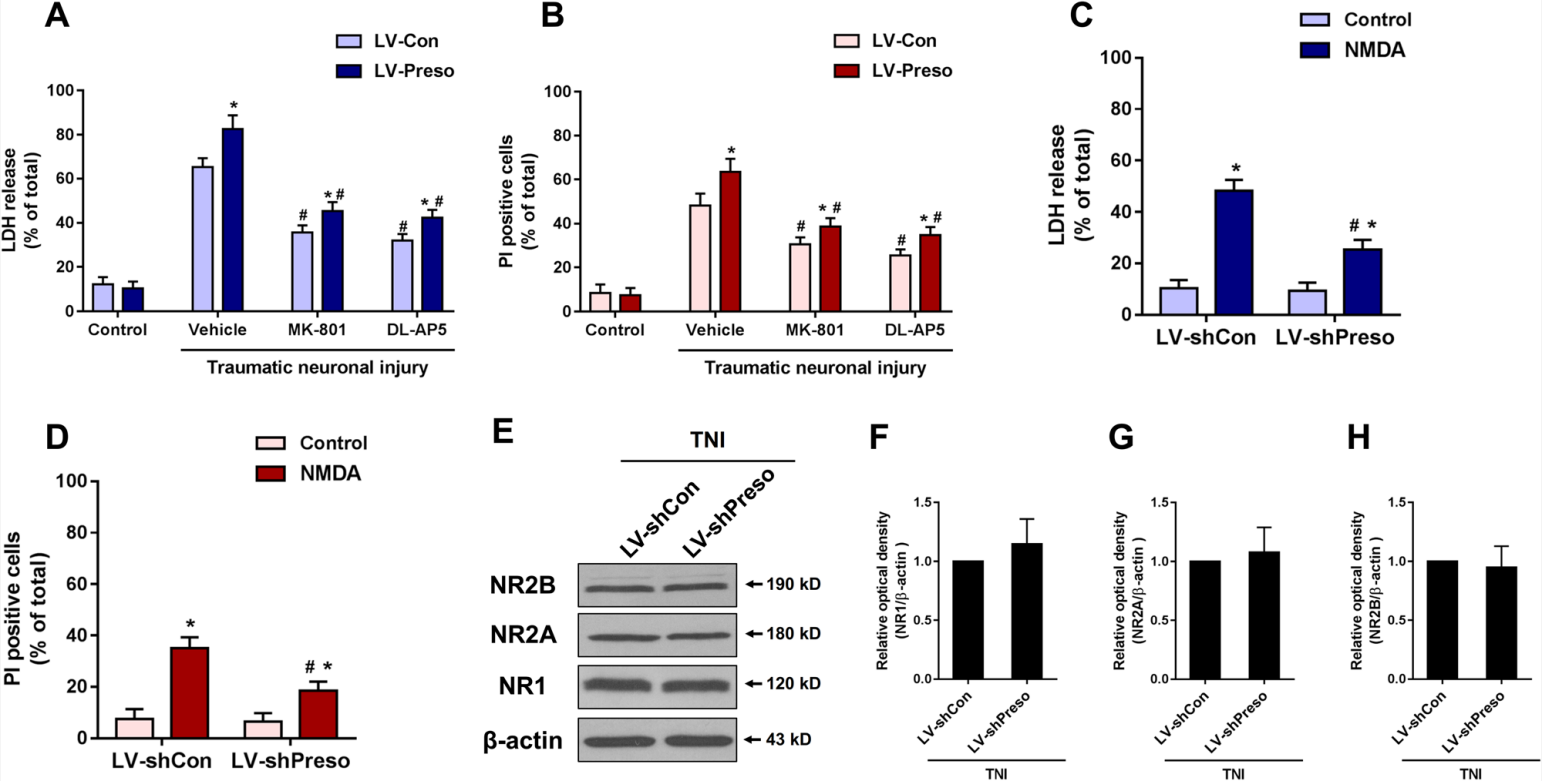
Preso regulates NMDAR-mediated excitotoxicity. Mice cortical neuronal cultures were transfected with LV-Con and LV-Preso. After transfection, the neuronal cultures were pretreated with DL-AP5 (100 μM) and MK-801 (100 μM). Cytotoxicity and the cell death rate were analyzed in neuronal cultures after traumatic neuronal injury (**a**, **b**). The data are presented as the mean ± SEM from five experiments. *p < 0.05 *vs*. LV-Con and ^#^*p* < 0.05 *vs*. Vehicle. Mouse cortical neuronal cultures were transfected with LV-shCon and LV-shPreso. After transfection, the neuronal cultures were treated with NMDA (100 μM). Cytotoxicity and the cell death rate were analyzed in neuronal cultures after NMDA treatment (**c**, **d**). The data are presented as the mean ± SEM from five experiments. *p < 0.05 *vs*. LV-shCon and ^#^*p* < 0.05 *vs*. Con. After transfection of LV-shCon and LV-shPreso and TNI, the expression of NR1, NR2A, and NR2B was analyzed by western blot (E-H). The data are presented as the mean ± SEM from five experiments

Numerous studies have shown that glutamate receptor-mediated excitotoxicity plays crucial roles in TBI. To determine the effect of Preso on NMDAR-related excitotoxicity after TBI, cortical neurons were pretreated with the NMDAR-specific antagonists DL-AP5 (100 μM) and MK-801 (100 μM). Consistent with the data mentioned above, upregulation of Preso expression elevated the cell death rate and release of LDH at 12 h after TNI. These effects were prevented by pretreatment with DL-AP5 and MK-801 (Fig. 2a, b). Furthermore, NMDA was used to induce excitotoxicity in mouse cortical neurons. Downregulation of Preso expression by LV-shPreso reduced neuronal injury after NMDAR activation (Fig. 2c, d). However, alteration of Preso did not affect the expression of NMDAR subunits, including NR1, NR2A, and NR2B (Fig. 2e–h). These results suggested that Preso modulated the function of NMDAR by means other than affecting NMDAR expression.

### Preso regulated Ca^2+^/NO responses mediated by NMDAR-related excitotoxicity

**Fig. 3:**
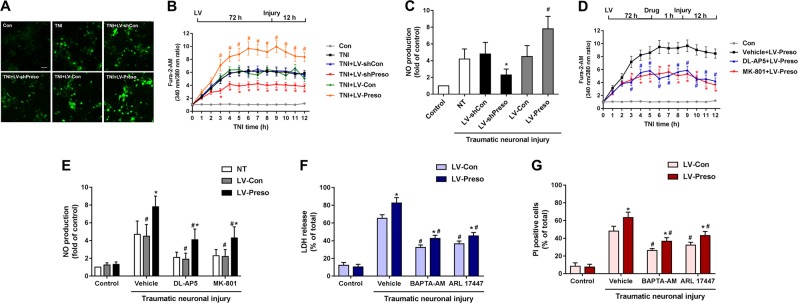
Regulation of NMDAR-related Ca^2+^/NO responses by Preso. Mouse cortical neuronal cultures were transfected with different lentiviruses. After transfection, the intracellular Ca^2+^ concentrations and NO production were analyzed after traumatic neuronal injury for 12 h (**a**-**c**). The data are presented as the mean ± SEM from five experiments. *p < 0.05 *vs*. LV-shCon and ^#^*p* < 0.05 *vs*. LV-Con. Scale bar = 50 μm. Mouse cortical neuronal cultures were transfected with LV-Con and LV-Preso. After transfection, the neuronal cultures were pretreated with DL-AP5 (100 μM) and MK-801 (100 μM). The intracellular Ca^2+^ concentrations and NO production were analyzed at 12 h after TNI (D-E). The data are presented as the mean ± SEM from five experiments. *p < 0.05 *vs*. LV-Con and ^#^*p* < 0.05 *vs*. Vehicle. After transfection, the neuronal cultures were pretreated with BAPTA-AM (10 μM) and ARL 17447 (20 μM). Cytotoxicity and the cell death rate were analyzed in neuronal cultures at 12 h after TNI (E-F). The data are presented as the mean ± SEM from five experiments. *p < 0.05 *vs*. LV-Con and ^#^*p* < 0.05 *vs*. Vehicle

One possible explanation for the modulation of NMDAR function by Preso might be related to calcium responses or subsequent NO generation. To determine the role of Preso in regulating Ca^2+^ mobilization and NO production after TNI, we used established probes for free Ca^2+^ (Fura-2a) and NO (DAF-FM). Downregulation of Preso expression inhibited Ca^2+^ overload and NO production, while upregulation of Preso expression elevated Ca^2+^ levels and NO responses (Fig. 3a–c). The effects of Preso upregulation on Ca^2+^ and NO responses were suppressed by pretreatment with the NMDAR antagonists DL-AP5 and MK-801 (Fig. 3d, e). To further clarify the role of Ca^2+^/NO responses in Preso-mediated neuronal injury, we used BAPTA-AM (10 μM) and ARL 17447 (20 μM) to eliminate intracellular Ca^2+^ and inhibit NO production. Pretreatment with BAPTA-AM and ARL 17447 reduced the elevation of LDH release and cell death induced by upregulation of Preso expression at 12 h after TNI (Fig. 3f, g). Therefore, these results indicate that Preso-mediated Ca^2+^/NO responses contribute to NMDAR-related excitotoxicity after TBI.

### Interaction between Preso and PSD-95 regulated the NMDAR-induced NO response

**Fig. 4:**
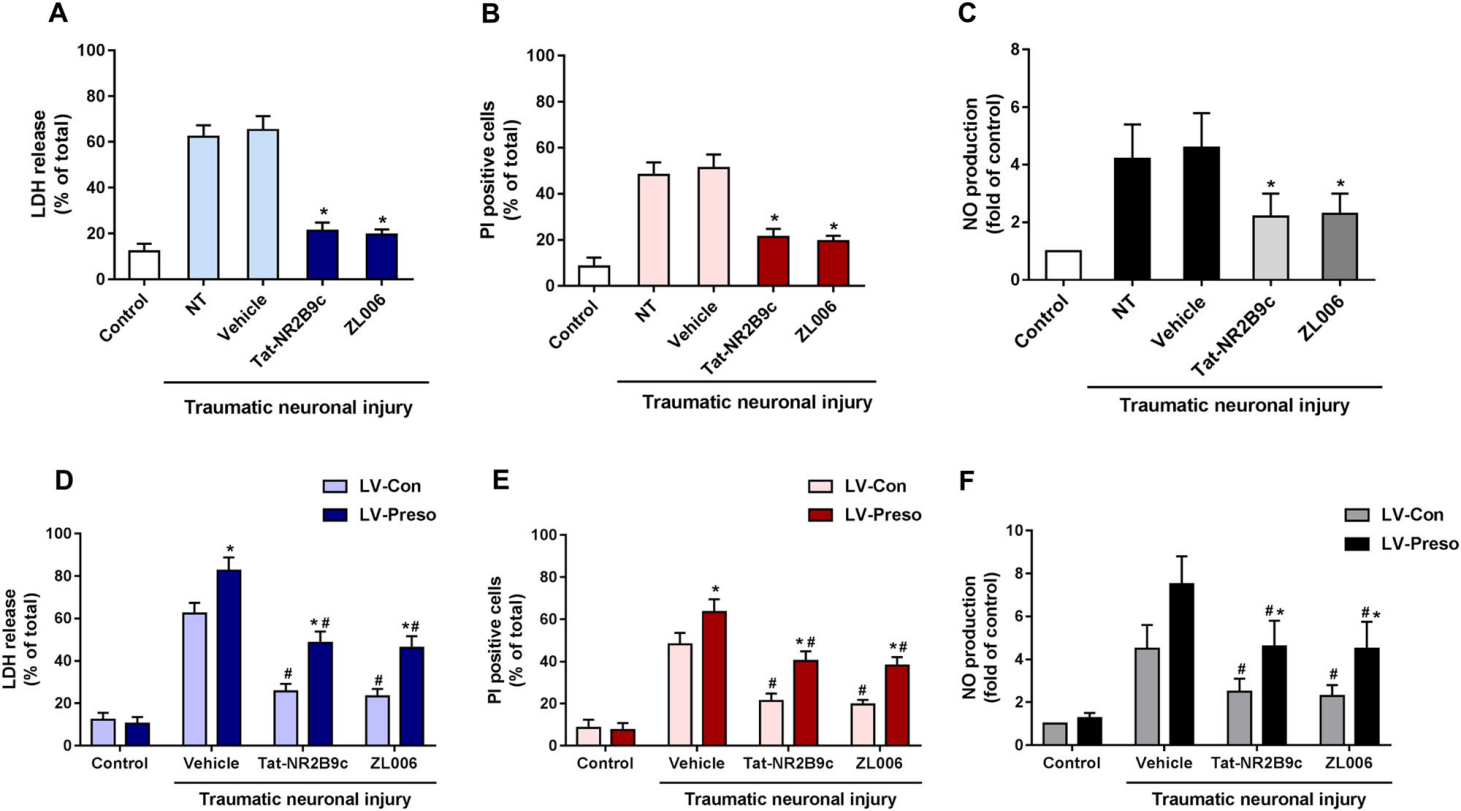
PSD-95 mediates the Preso regulation of the NO response. Mouse cortical neuronal cultures were pretreated with Tat-NR2B9c (10 μM) and ZL006 (10 μM). The cytotoxicity, cell death rate, and NO production in neuronal cultures were analyzed after traumatic neuronal injury (**a**-**c**). The data are presented as the mean ± SEM from five experiments. *p < 0.05 *vs*. Vehicle. Mouse cortical neuronal cultures were transfected with LV-Con and LV-Preso. After transfection, the neuronal cultures were pretreated with Tat-NR2B9c (10 μM) and ZL006 (10 μM). The cytotoxicity, cell death rate, and NO production in neuronal cultures were analyzed at 12 h after TNI (**d**-**f**). The data are presented as the mean ± SEM from five experiments. *p < 0.05 *vs*. LV-Con and ^#^*p* < 0.05 *vs*. Vehicle

PSD-95 is an important scaffold protein that facilitates signaling from NMDAR to NO by formation of the NR2B/PSD-95/nNOS complex. To investigate the role of PSD-95 in Preso-mediated neuronal injury and NO response, we used two antagonists specific for PSD-95, Tat-NR2B9c (to disrupt the interaction between NR2B and PSD-95) and ZL006 (to disrupt the interaction between PSD-95 and nNOS). As expected, Tat-NR2B9c (10 μM) and ZL006 (10 μM) reduced the neurotoxicity and inhibited the NO response at 12 h after TNI (Fig. 4a–c). These two drugs also suppressed the increased LDH release, cell death, and NO production induced by upregulated Preso expression following TNI (Fig. 4d–f).

**Fig. 5:**
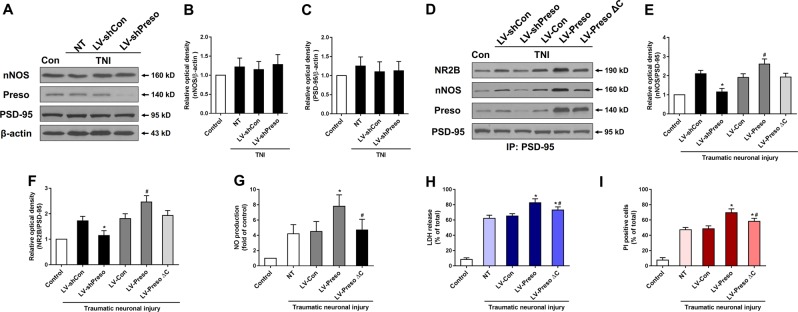
Interaction between Preso and PSD-95 regulates the NMDAR-induced NO response and excitotoxicity. Mouse cortical neuronal cultures were transfected with LV-shCon and LV-shPreso. After traumatic neuronal injury, the expression of PSD-95 and nNOS was analyzed by western blot (**a**-**c**). The data are presented as the mean ± SEM from five experiments. *p < 0.05 *vs*. LV-shCon. Mouse cortical neuronal cultures were transfected with different lentiviruses. After TNI, immunoprecipitants were obtained by PSD-95 immunoprecipitation. The expression of NR2B and nNOS was analyzed by western blot (**d**-**f**). The data are presented as the mean ± SEM from five experiments. *p < 0.05 *vs*. LV-shCon and ^#^*p* < 0.05 *vs*. LV-Con. The cytotoxicity, cell death rate, and NO production in neuronal cultures were analyzed at 12 h after TNI (**g**-**i**). The data are presented as the mean ± SEM from five experiments. *p < 0.05 *vs*. LV-Con and ^#^*p* < 0.05 *vs*. LV-Preso

To further clarify the mechanism by which Preso regulates the NR2B/PSD-95/nNOS complex, we assessed the role of Preso in modulating protein expression and the protein-protein interactions of the NR2B/PSD-95/nNOS complex. Similar to those of NR2B, the expression levels of PSD-95 and nNOS were not altered by upregulation of Preso expression (Fig. 5a–c). However, downregulation of Preso expression disrupted the interactions between PSD-95/NR2B and PSD-95/nNOS after excitotoxicity. In contrast, upregulation of Preso expression enhanced the PSD-95/NR2B and PSD-95/nNOS interactions (Fig. 5d–f). Because previous studies have shown that Preso has a PSD-95 binding site at its C-terminus, we constructed a lentiviral vector containing the Preso mutant variant lacking the PSD-95-binding C-terminus (LV-Preso ΔC). After deletion of the C-terminal PDZ domain, Preso did not increase the PSD-95/NR2B and PSD-95/nNOS interactions (Fig. 5d–f), and its effects on the NO response and neurotoxicity were also significantly reduced (Fig. 5g–i). These results indicated that Preso regulated the NMDAR-induced NO response by influencing the NR2B/PSD-95/nNOS complex.

### Involvement of CDK5 in the Preso regulation of the NMDAR-related Ca^2+^ response

**Fig. 6:**
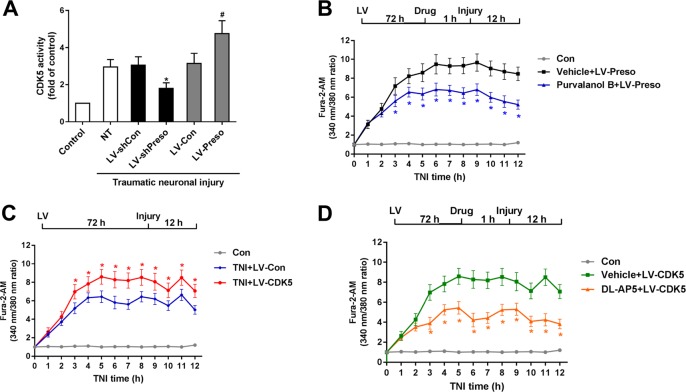
Involvement of CDK5 in the Preso regulation of the NMDAR-related Ca^2+^ response. Mouse cortical neuronal cultures were transfected with different lentiviruses. CDK5 activity was analyzed after traumatic neuronal injury (**a**). The data are presented as the mean ± SEM from five experiments. *p < 0.05 *vs*. LV-shCon and ^#^*p* < 0.05 *vs*. LV-Con. After transfection with LV-Preso, the neuronal cultures were pretreated with purvalanol B (50 μM). The intracellular Ca^2+^ concentrations were analyzed at 12 h after TNI (**b**). The data are presented as the mean ± SEM from five experiments. *p < 0.05 *vs*. Vehicle. Mouse cortical neuronal cultures were transfected with LV-Con and LV-CDK5. The intracellular Ca^2+^ concentrations were analyzed at 12 h after TNI (**c**). The data are presented as the mean ± SEM from five experiments. *p < 0.05 *vs*. LV-Con. After transfection, the neuronal cultures were pretreated with DL-AP5 (100 μM). The intracellular Ca^2+^ concentrations were analyzed at 12 h after TNI (**d**). The data are presented as the mean ± SEM from five experiments. *p < 0.05 *vs*. Vehicle

CDK5 has been shown to regulate the function of NMDAR and the Ca^2+^ response after brain injury. To examine the relationship between Preso and CDK5 after traumatic neuronal injury, cortical neurons were transfected with LV-Preso, LV-shPreso, and their control viruses. Upregulation of Preso expression increased CDK5 activity, while downregulation of Preso expression decreased CDK5 activity (Fig. 6a). After treatment with purvalanol B (50 μM), a CDK5 antagonist, the neuronal injury and Ca^2+^ overload induced by upregulated Preso expression were suppressed at 12 h after TNI (Fig. 6b).

**Fig. 7:**
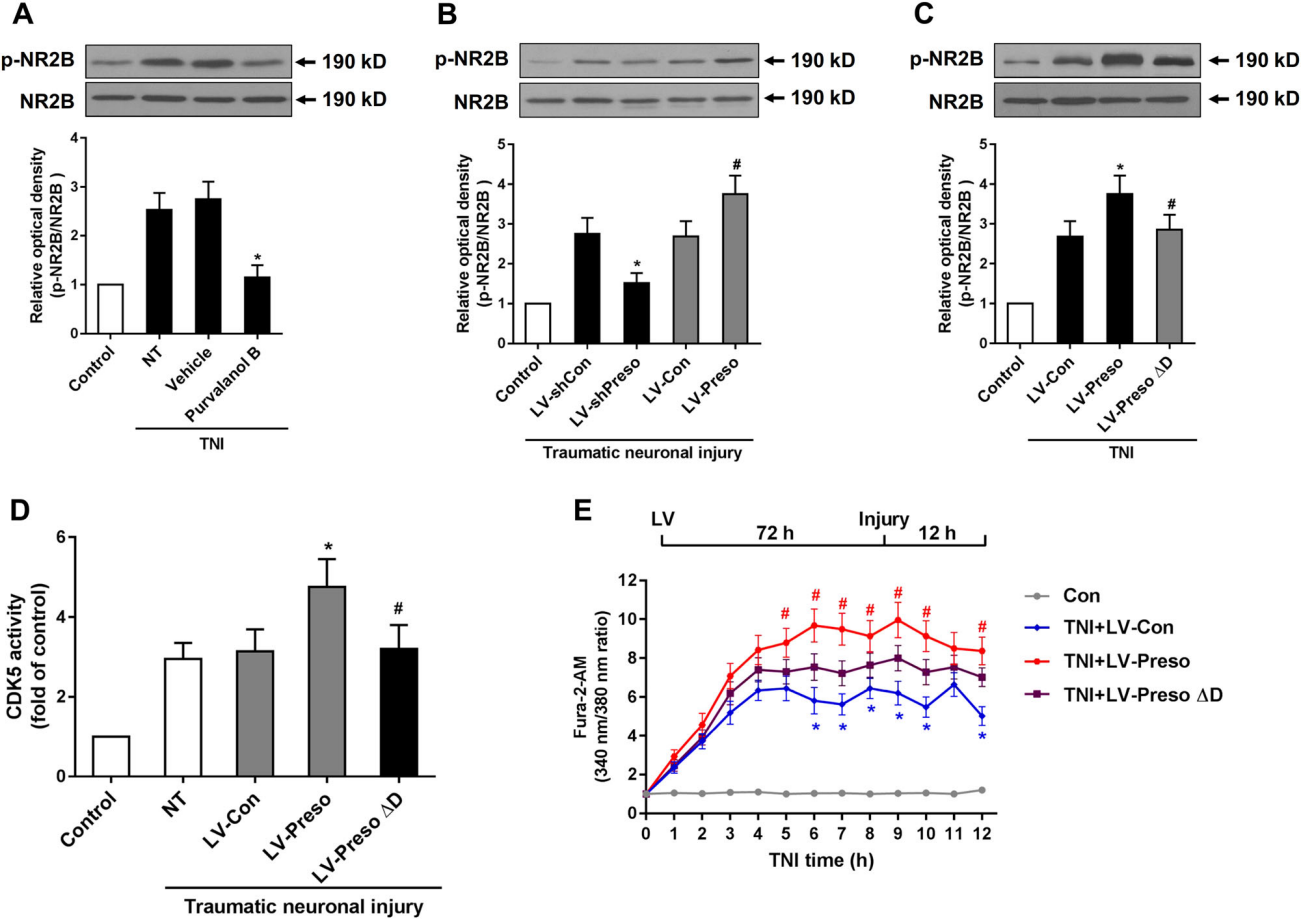
Preso regulates NR2B phosphorylation by modulating CDK5 activity. Mouse cortical neuronal cultures were pretreated with purvalanol B (50 μM). After traumatic neuronal injury, the phosphorylation of NR2B at Ser1284 was analyzed by western blot (**a**). The data are presented as the mean ± SEM from five experiments. *p < 0.05 *vs*. Vehicle. Mouse cortical neuronal cultures were transfected with different lentiviruses. NR2B phosphorylation was analyzed by western blot at 12 h after TNI (**b**). The data are presented as the mean ± SEM from five experiments. *p < 0.05 *vs*. LV-shCon and ^#^*p* < 0.05 *vs*. LV-Con. After transfection with LV-Preso ΔD, the NR2B phosphorylation, CDK5 activity, and intracellular Ca^2+^ concentration were analyzed at 12 h after TNI (**c**-**e**). The data are presented as the mean ± SEM from five experiments. *p < 0.05 *vs*. LV-Con and ^#^*p* < 0.05 *vs*. LV-Preso

Next, we determined the role of CDK5 in the NMDAR-related Ca^2+^ response after TNI. After the transfection of lentivirus expressing CDK5 (LV-CDK5), the Ca^2+^ response induced by TNI was increased (Fig. 6c). This effect was reduced by the administration of DL-AP5 (100 μM), an NMDAR antagonist (Fig. 6d). Inhibition of CDK5 activity by purvalanol B reduced the phosphorylation of NR2B at S1284 at 12 h after TNI (Fig. 7a). Downregulation of Preso expression reduced the phosphorylation of NR2B, while upregulation of Preso expression enhanced the phosphorylation of NR2B (Fig. 7b). Because Preso contains a D-domain, which acts as a binding site for proline-directed kinase, we constructed a lentiviral vector containing the Preso mutant variant lacking the D-domain (LV-Preso ΔD). After deletion of the D-domain, the effects of Preso on the phosphorylation of NR2B and CDK5 activity were significantly attenuated (Fig. 7c, d), and its effects on the Ca^2+^ response were also suppressed (Fig. 7e). These results suggested that the NMDAR-related Ca^2+^ response was modulated by Preso through its interaction with CDK5.

### Downregulation of Preso expression improved recovery after TBI

**Fig. 8:**
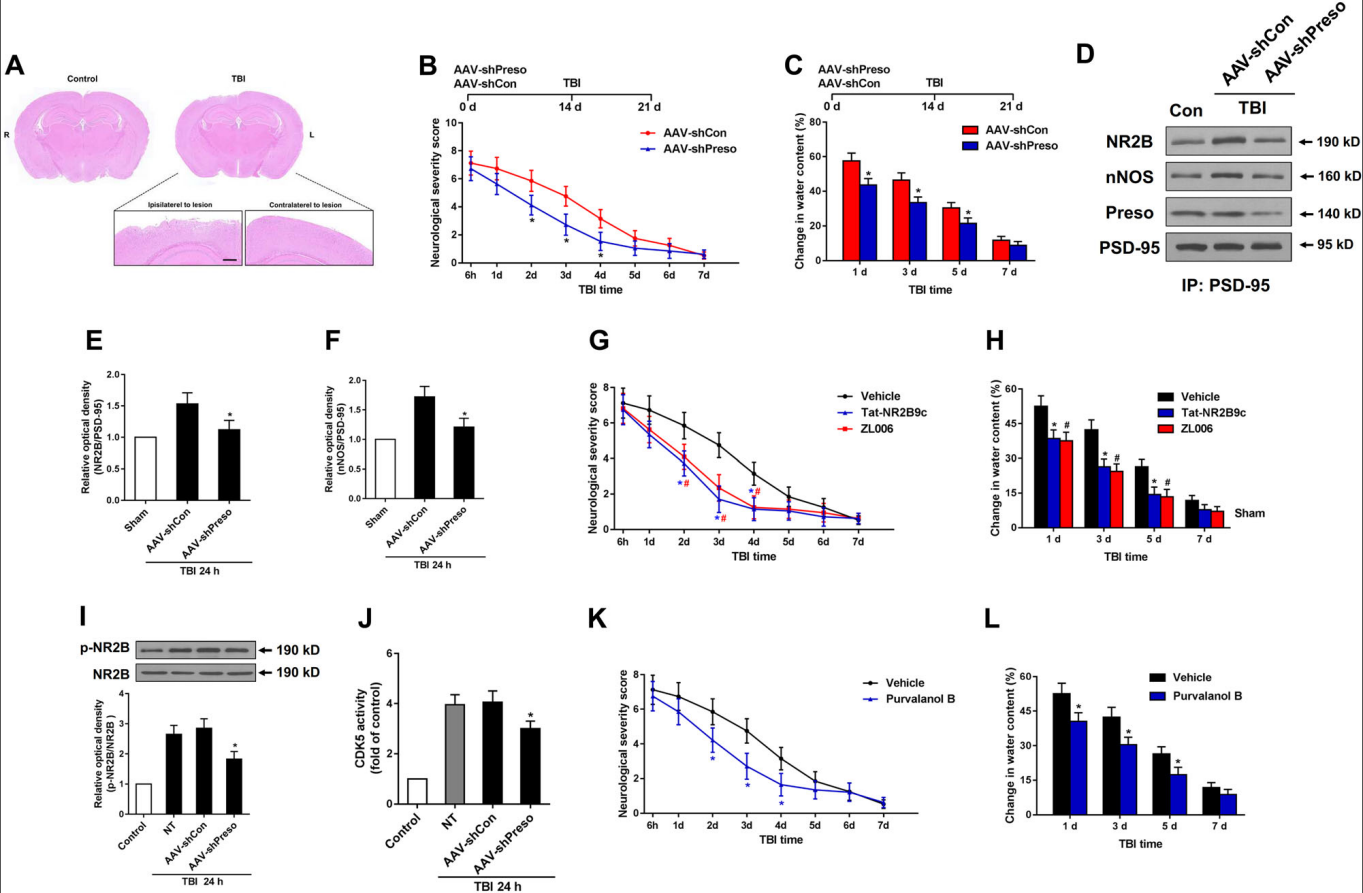
Downregulation of Preso expression protects against TBI. Mice were injected with AAV-shCon or AAV-shPreso, and TBI was then induced. H&E staining was performed on traumatically injured brain tissue (**a**). Scale bar = 200 μM. Neurological functions in the AAV-shCon group (*n* = 10) and AAV-shPreso group (*n* = 10) were assessed by determining the NSS at 6 h and at 1, 2, 3, 4, 5, 6, and 7 d after TBI (**b**). The extent of brain edema in these groups was investigated by change in water content at 1, 3, 5, and 7 d after TBI (**c**). The data are presented as the mean ± SEM. *p < 0.05 *vs*. AAV-shCon. Immunoprecipitants were obtained by PSD-95 immunoprecipitation. The expression of Preso, PSD-95, NR2B, and nNOS and phosphorylated NR2B were analyzed by western blot (**d**-**f**, **i**). The data are presented as the mean ± SEM from five experiments. *p < 0.05 *vs*. AAV-shCon. After transfection, the mice were treated with different drugs. The NSS at 6 h and at 1, 2, 3, 4, 5, 6, and 7 d after TBI and the extent of brain edema at 1 d, 3 d, 5 d, and 7 d after TBI were analyzed (**g**-**h**, **k**-**l**). The data are presented as the mean ± SEM from five experiments. *p < 0.05 *vs*. Vehicle

To further clarify whether Preso has similar effects in vivo, we used a controlled cortical impact (CCI) model to mimic TBI in vivo (Fig. 8a). We infused adeno-associated viruses containing shRNA Preso (AAV-shPreso) into the unilateral cortices of mice (Fig. S2). Two weeks later, brain trauma was induced in the cortex ipsilateral to the AAV-shPreso or AAV-shCon injection site, and the brain water content was measured at 1, 3, 5, and 7 d after TBI. The mouse neurological severity scores (NSSs) were assessed for seven consecutive days after injury. Downregulation of Preso expression in the lesion hemisphere significantly reduced brain edema and neurological deficits (Fig. 8b, c). Similar to data obtained from the in vitro studies, interactions of PSD-95/NR2B and PSD-95/nNOS were disrupted in lesions infected by AAV-shPreso (Fig. 8d–f). Disruption of PSD-95/NR2B and PSD-95/nNOS by Tat-NR2B9c and ZL006, respectively, improved recovery after TBI (Fig. 8g, h). NR2B phosphorylation and CDK5 activity was reduced by the administration of AAV-shPreso (Fig. 8i, j). Inhibition of CDK5 activity by purvalanol B attenuated brain edema and neurological deficits (Fig. 8k, l). Overall, these results suggested that positive regulation of the NR2B/PSD-95/nNOS complex and CDK5 activity by Preso plays an important role in brain damage after TBI.

## Discussion

Postsynaptic scaffold proteins act as crucial coordinators of synaptic function due to their interaction with synaptic receptors and their intracellular mediators. As a postsynaptic scaffold protein with FERM, WW, Homer ligand, and PDZ domains, Preso directly links with G protein-coupled receptors (mGluR), other scaffold proteins (PSD-95 and Homer), PAK-interacting exchange factor-beta (βPix), membrane phosphatidylinositol 4,5-bisphosphate (PIP2), proline-directed kinases (CDK5 and ERK), and the cytoskeleton and has been reported to regulate dendritic spine morphogenesis and the inflammatory pain response^5,6,8,9^. Our present study showed that inhibition of Preso expression suppressed neuronal injury and prevented brain damage after TBI, demonstrating that downregulating Preso expression exerts an incremental neuroprotective effect on TBI. This effect of Preso on TBI might be associated with its role in regulating excitotoxicity. Accordingly, the ability of Preso to mediate excitotoxicity raises the question of whether a similar Preso function is involved in other mechanisms following TBI.

Glutamate receptors play an important role in regulating neuronal death and survival following TBI. Postsynaptic scaffolds constitute a molecular framework at the postsynaptic level that provides a platform for synaptic transmission related to glutamate receptors. Therefore, the interaction between glutamate receptors and postsynaptic scaffold proteins contributes to the modulation of neuronal injury after TBI. Disruption of the interaction between PSD-95 and NR2B, a NMDAR subunit, reduced brain damage and improved neurological dysfunction after TBI^4,10^. Furthermore, Homer 1a attenuated neuronal injury and brain damage after TBI by differentially regulating group I mGluRs^11^. Our previous study showed that Preso affects neuronal injury related to NMDAR activation following glutamate-induced excitotoxicity^7^. After investigating the relationship between Preso and glutamate receptors in TBI, we herein showed that Preso serves as a crucial mediator of NMDAR signaling, which is in agreement with our previous study on excitotoxic neuronal injury.

The overload of intracellular Ca^2+^ by excessive NMDAR activation is regarded as the key mechanism underlying secondary brain injury following TBI^12^. This regulation of Ca^2+^ redistribution is related to the ion channel function of NMDAR at the postsynaptic membrane. The NMDAR-induced overload of Ca^2+^ participates in the activation of intracellular signaling pathways responsible for neuronal injury and dysregulation of synaptic plasticity. Furthermore, activation of NMDAR induces the overproduction of NO, resulting in nitrative stress and mitochondrial dysfunction after TBI^13^. This process is associated with modulation of nNOS by NMDAR activation^14^. In a previous study, we confirmed that Preso regulates NMDAR-related Ca^2+^ mobilization, suggesting that it exerts regulatory effects on the ion channel function of NMDAR^7^. In the present study, we further affirmed that Preso promotes not only Ca^2+^ overload but also NO overproduction by regulating NMDAR after TBI. Therefore, it is important to further clarify the mechanism by which Preso modulates NMDAR-induced Ca^2+^/NO responses in TBI.

Postsynaptic signaling transduction from NMDAR to NO is dependent on formation of the NR2B/PSD-95/nNOS complex^15–17^. Recently, uncoupling the formation of the NR2B/PSD-95/nNOS complex with pharmacological inhibitors has shown a promising neuroprotective effect^18–20^. In our previous study, regulation of Preso did not change the expression of the NMDAR subunits or influence their surface distribution, demonstrating that Preso may not be related to the expression and trafficking of NMDAR^7^. Like NR2B, Preso also did not affect the expression of PSD-95 and nNOS in the present study (Fig. S3). Overall, the regulatory effects of Preso on TBI via the NR2B/PSD-95/nNOS complex are not associated with changes in the components of this complex. Our subsequent study showed that downregulation of Preso expression disrupts the NR2B/PSD-95/nNOS complex by reducing the interaction of PSD-95 with NR2B and the interaction of PSD-95 with nNOS. Structurally, the C-terminal PDZ binding domain of Preso provides a binding site for PSD-95^5^. Compared with Preso without any mutations, the C-terminal mutant of Preso did not affect the formation of the NR2B/PSD-95/nNOS complex, suggesting that the binding of Preso to PSD-95 plays a crucial role in NR2B/PSD-95/nNOS complex regulation. This phenomenon of Preso promoting the formation of the NR2B/PSD-95/nNOS complex might be associated with its ability to enhance the stability of PSD-95 at the postsynaptic level.

CDK5 is a proline-directed serine/threonine kinase that plays an important role in synaptic plasticity and neurological diseases^21^. Aberrant CDK5 activity has been shown to be a principle cause of neuronal cell death during TBI^22^. Consistent with a previous study, we found that Preso positively regulated CDK5 activity, contributing to the neurotoxicity and overload of Ca^2+^ after traumatic injury. Recent studies have reported that NMDAR phosphorylation exerts various regulatory effects on receptor function, including synaptic trafficking and Ca^2+^ permeability^23,24^. CDK5 has been implicated in the phosphorylation of certain serine sites on NMDARs. Phosphorylation of NR2A at serine 1232 by CDK5 leads to enhanced NMDAR function in neuronal cell death^25^. CDK5 modulates the surface expression of NR2B and memory formation through its phosphorylation of NR2B at serine 1116^26^. In the present study, we found that Preso modulated the phosphorylation of NR2B at serine 1284 by regulating CDK5. Although this NR2B phosphorylation site was previously reported^27,28^, its role in TBI remained unclear. Thus, we demonstrated that the phosphorylation of NR2B at serine 1284 is tightly associated with the NMDAR-induced Ca^2+^ response after TBI. This regulatory pattern was shown to be dependent on the Preso D-domain, which is a binding site for proline-directed kinases^6^.

**Fig. 9:**
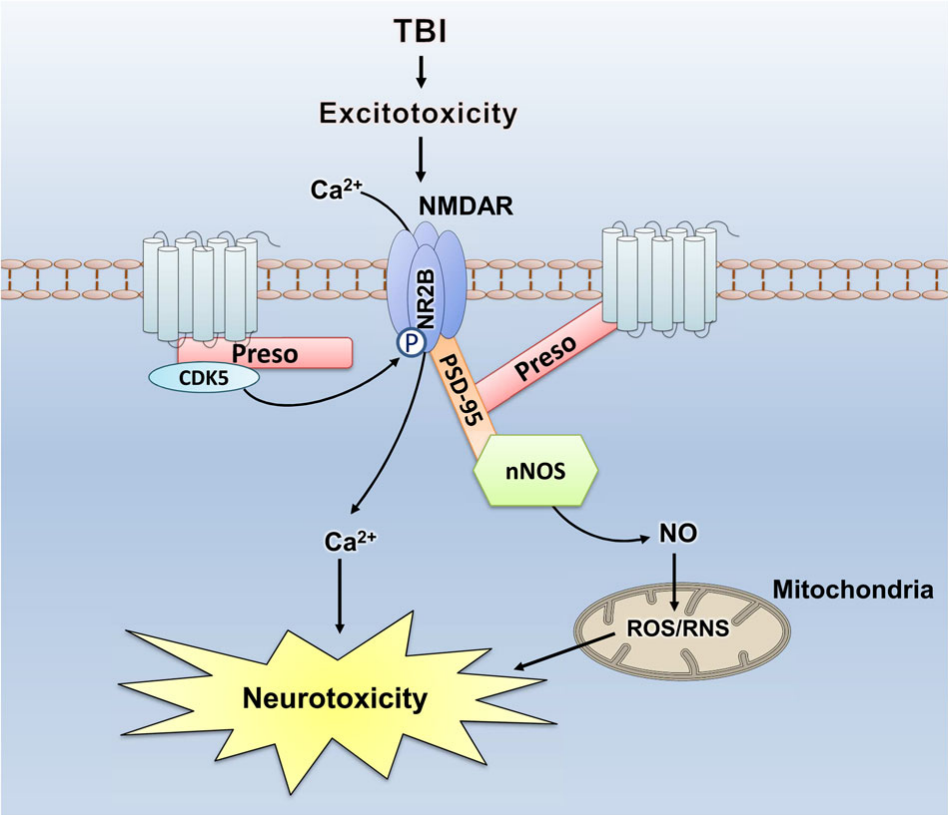
A proposed diagram tying together the various observations involved in the regulation of NMDAR signaling by Preso after TBI. *TBI* traumatic brain injury, *NMDAR* N-methyl-D-aspartate receptor, *PSD-95* postsynaptic density-95, *CDK5* cyclin-dependent kinase 5, *nNOS* neuronal nitric oxide synthase, *Ca*^*2+*^ calcium, *NO* nitric oxide, *ROS* reactive oxygen species, *RNS* reactive nitrogen species

In the present study, we elucidated the mechanism by which Preso modulates NMDAR-related excitotoxicity after TBI (Fig. 9). However, the role of Preso in TBI and its underlying mechanisms may not be limited to the regulation of NMDAR signaling. In previous studies, mGluR signaling has been reported to be another important target of Preso in the regulation of synaptic function. Preso binds mGluR via its FERM domain and contains a Homer protein binding site^6,9^. Because our previous studies showed the crucial effects of mGluR and Homer on TBI, Preso is expected to regulate TBI via its interaction with mGluR-Homer signaling. Moreover, the characteristic structure of Preso provides various protein-protein interaction patterns and targets for interfering with different intracellular signaling pathways. Compounds targeting these binding sites might accurately regulate Preso and its downstream mediators, thereby specifically interfering with synaptic function and neurological diseases. Therefore, further investigation is required to elucidate the mechanism of Preso in neurological diseases and elucidate novel targets for these diseases.

## Materials and methods

### Animals

C57BL/6 mice (10–12 weeks, 25–28 g), obtained from the Experimental Center of Fourth Military Medical University, were maintained at a constant temperature (approximately 27 °C) in an air-conditioned room for at least 7 d before the study and exposed to a 12-h light/dark cycle. All animal studies were performed in adherence with the National Institutes of Health Guidelines for the Care and Use of Laboratory Animals and approved by the Fourth Military Medical University Committee on Animal Care.

### Primary culture of cortical neurons

Neuronal cortical cultures were prepared as previously described with some modifications^29^. Briefly, cerebral cortices were removed from embryos at 16-18 d. Tissues were dissociated by 0.25% trypsin for 15 min at 37 °C and gentle trituration. Neurons were resuspended in neurobasal medium containing 2% B27 supplement and 0.5 mM L-glutamine (Thermo Fisher Scientific, Rockford, IL, USA) and plated at a density of 3 × 10^5^ cells/cm^2^. Before seeding, culture vessels consisting of 96-well plates, 1.5-cm glass slides or 6-cm dishes were coated with poly-L-lysine (50 μg/mL) at room temperature overnight. The neurons were maintained at 37 °C in a humidified 5% CO_2_ incubator, and half of the culture medium was changed every other day. The cultured neurons were used for in vitro studies on days 12-14 (DIV 12-14) and verified to be greater than 95% viable.

### Antibodies and reagents

A primary antibody against Preso was obtained from R&D Systems, Inc. (Minneapolis, MN, USA). Antibodies against nNOS, NR1, NR2A, NR2B, and PSD-95 were obtained from NeuroMab (Davis, CA, USA). Antibodies against CDK5 and phospho-NR2B (Ser1284) were obtained from Cell Signaling Technology (Danvers, MA, USA). An antibody against β-actin was obtained from Sigma-Aldrich (St. Louis, MO, USA). For immunoblotting, HRP-conjugated anti-rabbit and anti-mouse secondary antibodies were used (Santa Cruz Biotechnology, CA, USA). The Alexa Fluor 488 mouse IgG and Alexa Fluor 594 rabbit IgG secondary antibodies (Thermo Fisher Scientific) were used for immunostaining. DL-AP5, MK-801, BAPTA-AM, ARL 17447, and purvalanol B were obtained from Tocris Bioscience (Bristol, UK). ZL006 was obtained from EMD Millipore (Billerica, MA, USA). Tat-NR2B9c was obtained from ProbeChem (St. Pete Beach, FL, USA).

### TBI models

The in vitro model of TBI employed in the present study was previously described by Mukhin et al. with some modification^29^. This TNI employed a plastic stylet to scrape adherent cells from a culture dish, thereby tearing processes and soma while leaving a significant proportion of cells intact. This model was employed in the present study as described previously^11^. Briefly, each confluent cell culture was manually scratched with a sterile plastic pipette tip following a square grid (with 3 mm spacing between the lines). To reduce the inconsistency of damage in different experiments, all TNI models were established by the same researcher in our group using a standard square grid module. The cultured neurons were used for in vitro studies on DIV 12-14. Culture cells were placed in an incubator at 37 °C until a designated posttrauma time point was reached, and the medium was not changed. Experiments were performed from 0 h (immediately after mechanical injury) to 24 h after trauma. Uninjured cell cultures were used as controls. Because scratch injury first activates neurons at the wound edge and later expands to the entire neuronal monolayer, the entire culture on each dish was used for all experiments.

A CCI model was used as the in vivo model of TBI in the present study. After induction of anesthesia with 4% isoflurane, a mouse was placed on a platform with a stereotactic frame. A midline longitudinal scalp incision was made, the skull was exposed, and the dura was kept intact. Injury was induced by impacting the right cortex with an actuator (3 mm diameter) at a rate of 3 m/s and a compression of 1.5 mm. Then, the scalp wound was closed with standard suture material, and the wound area was treated with lidocaine cream. The mice were returned to their cages at the end of the surgical procedures, where and water and food were available ad libitum.

### Preparation of lentivirus and adeno-associated virus for RNAi and overexpression

All lentiviruses and adeno-associated viruses were developed and obtained from GeneChem Company (Shanghai, China). To develop the shRNA lentiviruses and shRNA adeno-associated virus (AAV), an siRNA oligo (CCTTGTGTCCCAAAGAGCA) was subcloned into a GV248 lentiviral vector (hU6-MCS-Ubiquitin-EGFP-IRES-puromycin) (S1) and a GV478 AAV serotype 2 vector (U6-MCS-CAG-EGFP) (Fig. S2). To develop overexpression lentiviruses, cDNAs of Preso, Preso without the PSD-95-binding C-terminus (deletion of amino acids 1309-1312), CDK5, and Preso without the D-domain (deletion of amino acids 641-655) were subcloned into a G492 lentiviral vector (Ubi-MCS-3FLAG-CBh-gcGFP-IRES-puromycin) (Fig. S1). The cultured neurons were used for transfection on DIV 12-14. After transfection with different lentiviruses for 72 h, neurons were prepared for further experiments. The AAV vector was injected into the cortex using a stereotaxic instrument. At 14 d after the cortical injection of AAV, mice were prepared for brain trauma.

### Western blot analysis

After various treatments, mouse cortical neurons were washed with ice-cold phosphate-buffered saline (PBS) three times and lysed with buffer containing PhosSTOP protease inhibitor and phosphatase inhibitor mixture tablets (Roche Applied Bioscience, Indianapolis, IN, USA). The protein concentration in the supernatant was determined using a BCA protein kit. The proteins were separated on 10-15% and 10% SDS-PAGE gels and transferred to nitrocellulose membranes (Thermo Fisher Scientific). The membranes were soaked in a 5% nonfat milk solution comprising Tris-buffered saline and 0.05% Tween 20 (TBST) for 1 h at room temperature and then incubated overnight at 4 °C with the appropriate primary antibodies (Preso, 1:500 dilution; NR1, 1:500 dilution; NR2A, 1:500 dilution; NR2B, 1:500 dilution; PSD-95, 1:1000 dilution; β-actin, 1:2500 dilution). The membranes were washed in TBST and incubated for 1 h at room temperature with the secondary antibodies diluted in blocking buffer. Immunoreactivity was detected by the SuperSignal West Pico Chemiluminescent Substrate (Thermo Fisher Scientific). The optical densities of the bands were quantified using an image analysis system with ImageJ (National Institutes of Health, MA, USA).

### LDH assay

Cytotoxicity was determined by the release of LDH, a cytoplasmic enzyme released from cells and a marker of membrane integrity. LDH release into the culture medium was detected using an LDH cytotoxicity assay kit obtained from Cayman Chemical (Ann Abor, MI, USA) according to the manufacturer’s instructions. Briefly, 100 μl of supernatant from each well was collected in a new 96-well plate. LDH reaction solution was added to each well, and the mixture was incubated for 30 min at 37 °C with gentle shaking. The activity of LDH was calculated from the absorbance measured at a wavelength of 490 nm with a plate reader.

### Cell death assessment

Following the exposure of neuronal cultures to the various treatments, neuronal survival was quantified and presented as the percentage of cell death. Neuronal survival was determined by staining treated neuronal cultures with 5 µM Hoechst 33342 and 2 µM PI (Sigma-Aldrich) for 10 min. Cell culture plates were placed on the mechanical stage of a Nikon microscope, and photomicrographs were acquired by a blinded observer. The numbers of total and injured (PI-positive) cells were counted by automated computer-assisted software (Image-Pro Plus version 6.0, Media Cybernetics, Inc.). The raw counts were analyzed in Microsoft Excel to determine the percent cytotoxicity and conduct statistical analysis.

### Measurement of intracellular nitric oxide

Intracellular nitric oxide (NO) was estimated using 4-amino-5-methylamino-2′,7′-difluorofluorescein (DAF-FM) diacetate obtained from Molecular Probes (Eugene, OR, USA) according to the manufacturer’s instructions. Briefly, neurons were prepared on slides, and a DMSO stock solution was diluted at a concentration of 10 µM. After the various treatments, neurons were incubated with DAF-FM diacetate for 60 min at room temperature. Then, the cells were washed to remove excess probe and incubated with fresh medium for 30 min. Fluorescence data were acquired using a fluorescence plate reader at an excitation wavelength of 495 nm and an emission wavelength of 515 nm.

### Calcium imaging

The intracellular Ca^2+^ concentration was measured using the ratiometric calcium indicator Fura-2-AM (Thermo Fisher Scientific). Cultured neurons grown on glass slides were treated with 5 μM Fura-2-AM for 45 min before various treatments at room temperature. The neurons were then placed in an open-bath imaging chamber containing Dulbecco’s PBS (0.901 mM CaCl_2_, 0.493 mM MgCl_2_-6H_2_O, 2.67 mM KCl, 1.47 mM KH_2_PO_4_, 137.93 mM NaCl, and 8.06 mM Na_2_HPO_4_-7H_2_O, pH 7.2-7.4) supplemented with 20 mM glucose at ambient temperature. Using a Nikon inverted epifluorescence microscope, the neurons were excited at wavelengths of 345 and 385 nm, and fluorescence images were acquired at an emission wavelength of 510 nm. The images were acquired and analyzed with MetaFluor image processing software. The Ca^2+^ concentration values were then calculated, and nonspecific Ca^2+^ fluorescence was subtracted from each wavelength before the calculations were conducted.

### Coimmunoprecipitation (Co-IP)

To evaluate protein interactions, Co-IP experiments were performed using a Pierce™ Crosslink Magnetic IP/Co-IP Kit (Thermo Fisher Scientific) according to the manufacturer’s instructions. Cortical neurons were cultured in 100-mm dishes and then harvested in ice-cold lysis/wash buffer supplemented with a proteinase inhibitor cocktail (Roche Applied Bioscience). The lysate was centrifuged at 13,000 × *g* for 15 min at 4 °C. Protein concentrations in the extracts were determined using a BCA protein assay kit (Thermo Fisher Scientific). Magnetic beads were crosslinked with nonspecific mouse IgG (2 mg), anti-PSD-95 (NeuroMab) and anti-CDK5 (Cell Signaling Technology), and the beads were then washed two times with coupling buffer. The protein extracts were combined with the beads and incubated overnight at 4 °C. Following magnetic isolation, the precipitates were washed three times with wash buffer, eluted with elution buffer, neutralized with neutralization buffer, and prepared for western blot and kinase activity assays.

### CDK5 activity assay

After various treatments, immunoprecipitants were obtained by CDK5 immunoprecipitation. A CDK5 activity assay was performed using the ADP-Glo™ Kinase Assay Kit (Promega Corporation, WI, USA) according to the manufacturer’s protocol. Briefly, enzyme, substrate, ATP and inhibitors were diluted in Kinase Buffer. The inhibitor, enzyme, and substrate/ATP mixture were added to the 96-well plate and incubated at room temperature for 10 min. Then, ADP-Glo™ Reagent was added to each well of the plate, and the mixture was incubated at room temperature for 40 min. Next, Kinase Detection Reagent was added to each well of the plate, and the mixture was incubated at room temperature for 30 min. The results were acquired by measuring the luminescence with a plate-reading luminometer.

### Stereotaxic injection of lentivirus

The in vivo delivery of AAV-shPreso and Vector was carried out using stereotaxic cortical injection as previously described^11^. Three cortical injections were performed in the right hemisphere (ipsilateral to the lesion) as follows: point 1, 1.0 mm anterior to the bregma, 1.5 mm lateral, and 1.5 mm deep; point 2, 0 mm antero-posterior to the bregma, 1.5 mm lateral, and 1.5 mm deep; and point 3, 1.0 mm posterior to the bregma, 1.5 mm lateral, and 1.5 mm deep. All the target points were in the right hemisphere (ipsilateral to the lesion). Each injection contained 1.5 μl of 1 × 10^9^ TU/ml AAV suspension, which was injected at a rate of 0.2 μl/min. The needle was withdrawn over the course of 10 min. Fourteen days after the lentivirus injection, mice were subjected to brain trauma as described above.

### Neurological severity score (NSS)

The NSS, which is highly correlated with the severity of brain damage, was measured as previously described^30^. Neurological severity scoring was conducted by an investigator who was blinded to the experimental groups. The score consists of 10 individual neurological parameters, including tasks on motor function, alertness and physiological behavior. One point was awarded for the lack of a tested reflex or for the inability to perform a task, and no points were awarded for success. A maximal NSS of 10 points indicated severe neurological dysfunction, with failure of all tasks.

### Water content measurement

The extent of cerebral edema was evaluated by determining the tissue water content in the injured hemisphere as previously reported^31^. Briefly, mice were decapitated under deep anesthesia with 100 mg/kg pentobarbital. Their brains were quickly removed, and the hemispheres were separated in the sagittal plane. Coronal sections with a thickness of 3 mm were prepared from the area bordering the lesion. These sections were weighed (wet weight) and dried in a desiccator oven for 24 h at 95 °C. After weighing the dried sections (dry weight), the water contents of the cortices ipsilateral to the injury (Wi) and contralateral to the injury (Wc) were calculated using equation one as follows: Water content (g/g dry weight) = (wet weight – dry weight)/dry weight. Next, the difference between Wi and Wc was calculated via equation two as follows: % Change in water content = [(Wi-Wc) × 100]/Wc.

### Statistical analysis

All experiments were performed a minimum of three times. Statistical evaluation was performed with GraphPad Prism software, version 6.0 (GraphPad, San Diego, CA). Significant differences between experiments were assessed by univariate ANOVA (more than two groups) followed by Bonferroni’s multiple comparison or unpaired t tests (two groups).

## Supplementary information

Figure S1

Figure S2

Figure S3
