# Supplementary figures and images for: Preso regulates NMDA receptor-mediated excitotoxicity via modulating nitric oxide and calcium responses after traumatic brain injury
Source: Cell Death Dis. 2019 Jun 24;10(7):496. doi: 10.1038/s41419-019-1731-x (PMC6591282; doi:10.1038/s41419-019-1731-x)

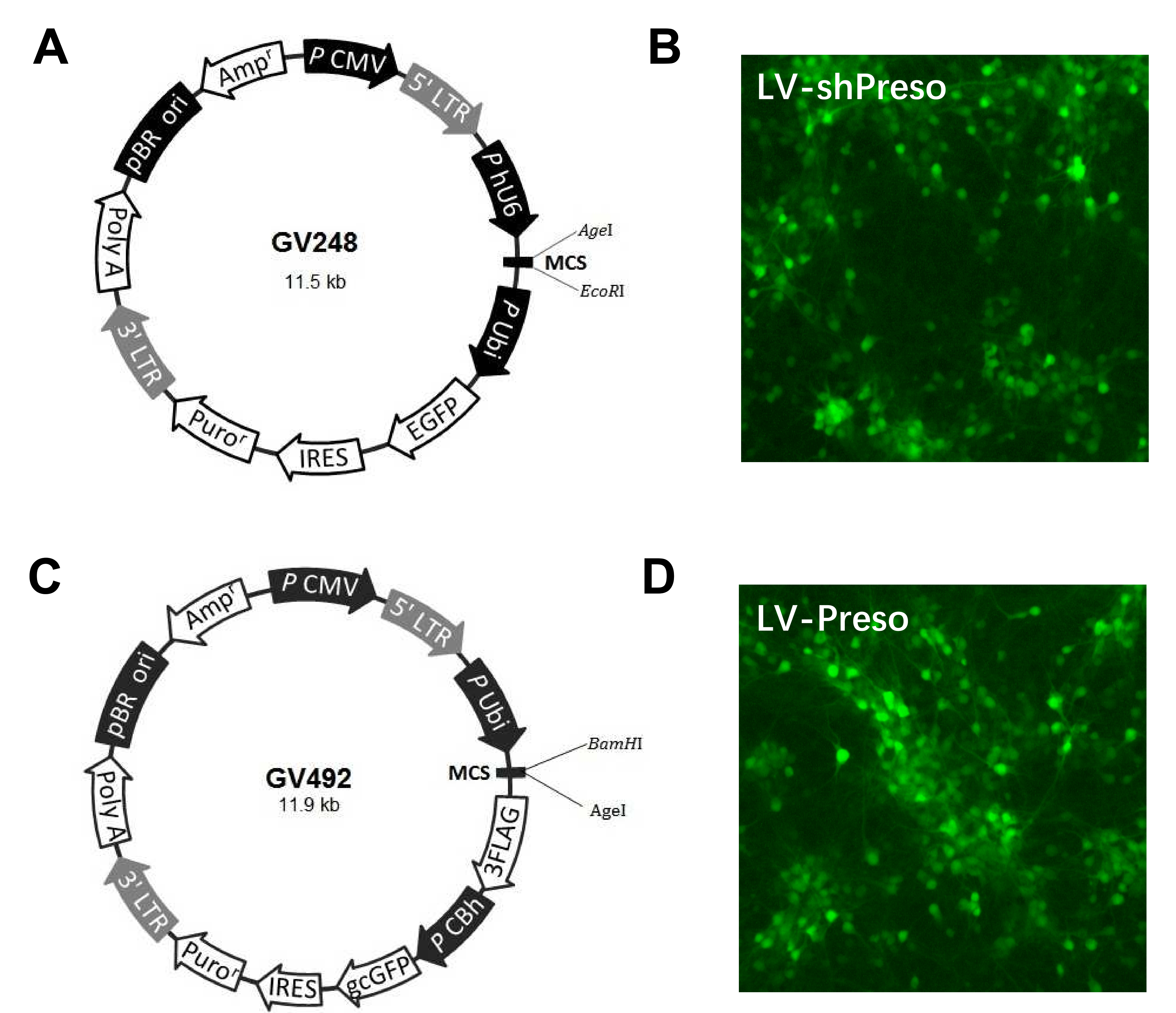

Supplement: Supplementary file 1 — Figure S1 [file 41419_2019_1731_MOESM1_ESM.tif]

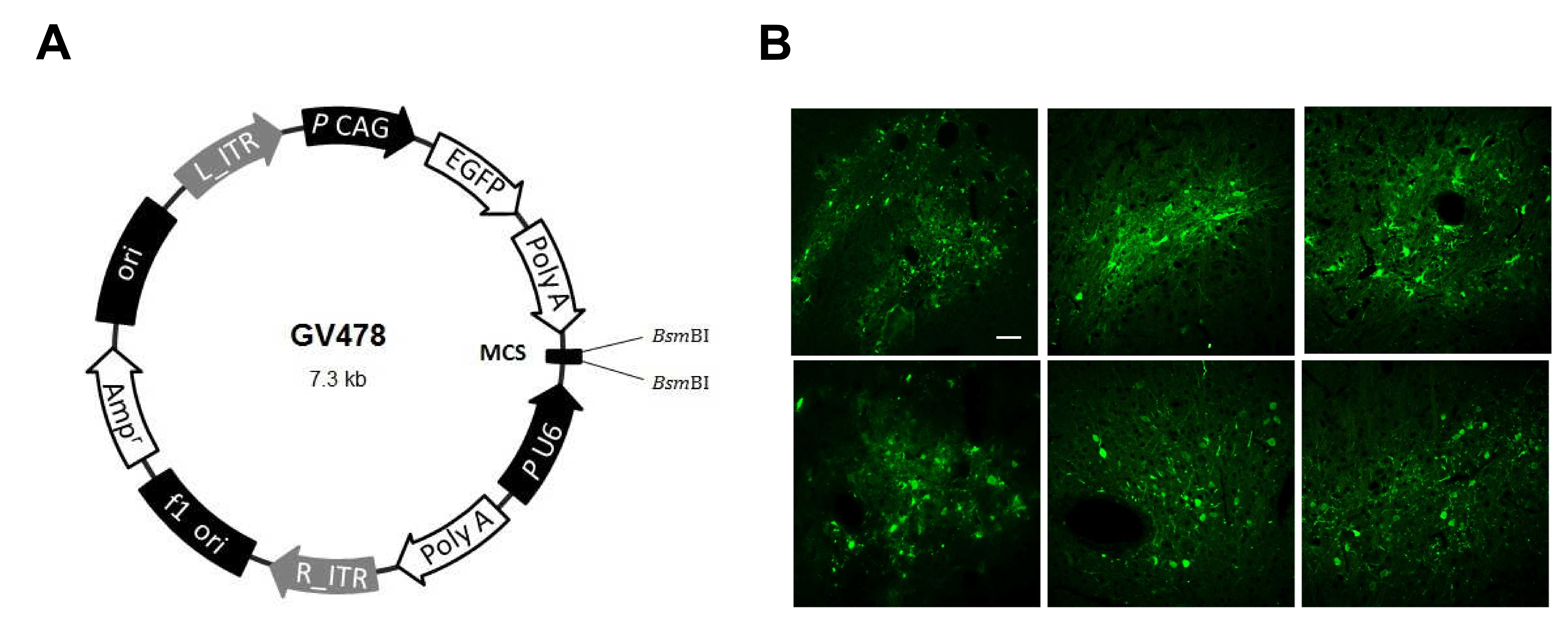

Supplement: Supplementary file 2 — Figure S2 [file 41419_2019_1731_MOESM2_ESM.tif]

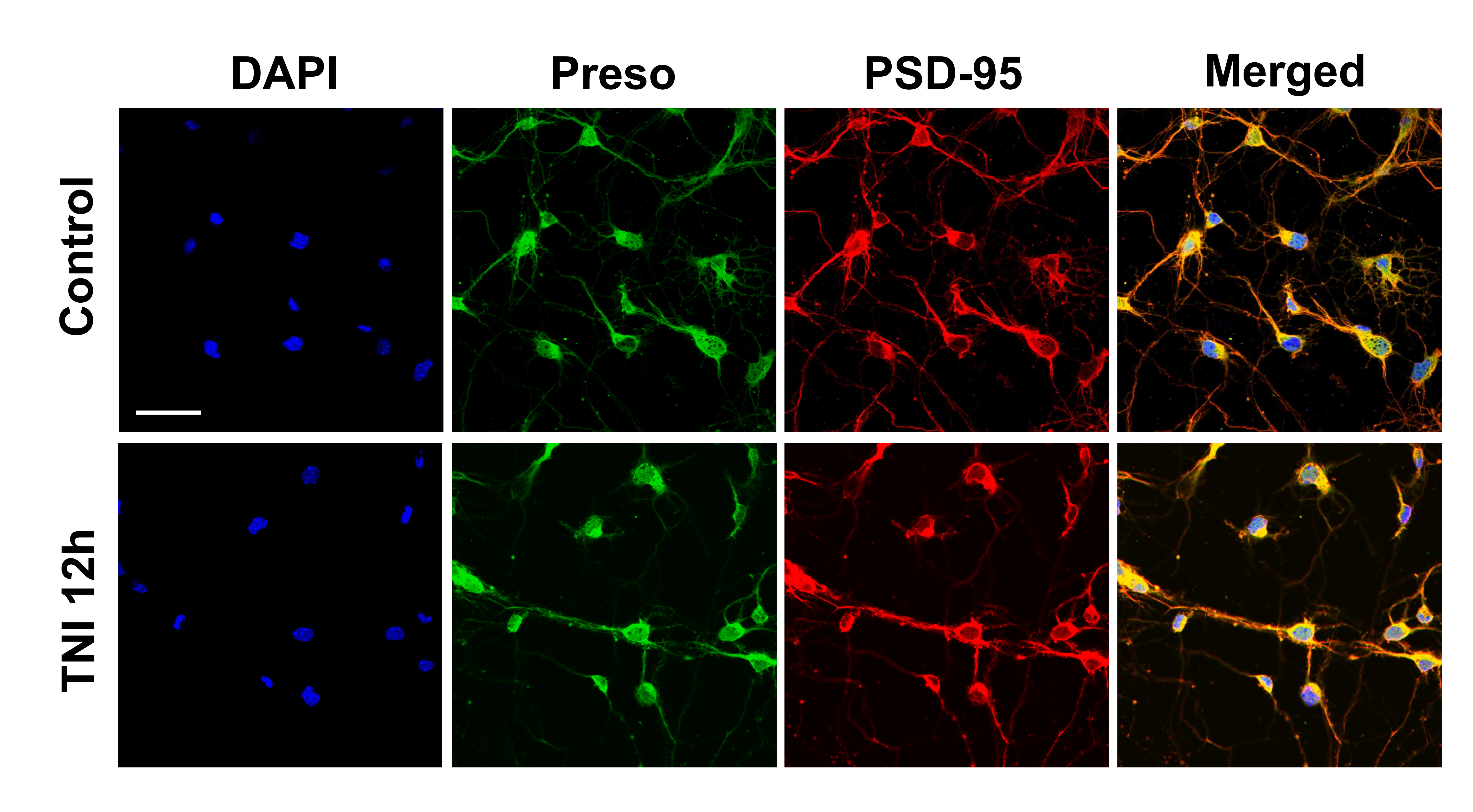

Supplement: Supplementary file 3 — Figure S3 [file 41419_2019_1731_MOESM3_ESM.tif]
